# Species-specific ant brain manipulation by a specialized fungal parasite

**DOI:** 10.1186/s12862-014-0166-3

**Published:** 2014-08-29

**Authors:** Charissa de Bekker, Lauren E Quevillon, Philip B Smith, Kimberly R Fleming, Debashis Ghosh, Andrew D Patterson, David P Hughes

**Affiliations:** Department of Entomology and Department of Biology, Center for Infectious Disease Dynamics, Pennsylvania State University, University Park, State College, Pennsylvania, PA 16802 USA; Metabolomics Core Facility, Huck Institutes of the Life Sciences, Pennsylvania State University, University Park, State College, Pennsylvania, PA 16802 USA; Department of Statistics and Public Health Sciences, Pennsylvania State University, University Park, State College, Pennsylvania, PA 16802 USA; Center for Molecular Toxicology and Carcinogenesis, Department of Veterinary and Biomedical Sciences, Pennsylvania State University, University Park, State College, Pennsylvania, PA 16802 USA

**Keywords:** Behavioral manipulation, Host specificity, Secretome, Metabolomics, *Ophiocordyceps unilateralis*

## Abstract

**Background:**

A compelling demonstration of adaptation by natural selection is the ability of parasites to manipulate host behavior. One dramatic example involves fungal species from the genus *Ophiocordyceps* that control their ant hosts by inducing a biting behavior. Intensive sampling across the globe of ants that died after being manipulated by *Ophiocordyceps* suggests that this phenomenon is highly species-specific. We advance our understanding of this system by reconstructing host manipulation by *Ophiocordyceps* parasites under controlled laboratory conditions and combining this with field observations of infection rates and a metabolomics survey.

**Results:**

We report on a newly discovered species of *Ophiocordyceps unilateralis sensu lato* from North America that we use to address the species-specificity of *Ophiocordyceps*-induced manipulation of ant behavior. We show that the fungus can kill all ant species tested, but only manipulates the behavior of those it infects in nature. To investigate if this could be explained at the molecular level, we used *ex vivo* culturing assays to measure the metabolites that are secreted by the fungus to mediate fungus-ant tissue interactions. We show the fungus reacts heterogeneously to brains of different ant species by secreting a different array of metabolites. By determining which ion peaks are significantly enriched when the fungus is grown alongside brains of its naturally occurring host, we discovered candidate compounds that could be involved in behavioral manipulation by *O. unilateralis s.l.*. Two of these candidates are known to be involved in neurological diseases and cancer.

**Conclusions:**

The integrative work presented here shows that ant brain manipulation by *O. unilateralis s.l.* is species-specific seemingly because the fungus produces a specific array of compounds as a reaction to the presence of the host brain it has evolved to manipulate. These studies have resulted in the discovery of candidate compounds involved in establishing behavioral manipulation by this specialized fungus and therefore represent a major advancement towards an understanding of the molecular mechanisms underlying this phenomenon.

**Electronic supplementary material:**

The online version of this article (doi:10.1186/s12862-014-0166-3) contains supplementary material, which is available to authorized users.

## Background

Parasites can adaptively manipulate host behavior resulting in complex extended phenotypes expressed in the bodies of their hosts that serve to increase parasite fitness [1]. While there are an increasing number of prominent examples of parasitic behavioral manipulation [2–6], the molecular mechanisms of host manipulation remain poorly understood [3–5,7–11]. One example where significant progress has been made is the parasite *Toxoplasma gondii.* Infected rodents, with parasite cysts within their neurons and glia [12], become more attracted to cat urine and display increased risk-taking behavior. These behaviors increase parasite transmission to the ultimate host, a feline [13]. Tyrosine hydroxylase secretion by *T. gondii* cysts increases the dopamine level inside neurons that harbor them [14]. However, increasing just dopamine levels in non-parasitized rodents does not result in similar behavioral changes [15]. This, together with research demonstrating that *T. gondii* also alters the levels of other compounds in infected rodents [16,17], suggests that multiple mechanisms are involved in establishing complex behavioral manipulation.

Another dramatic example of a parasite extended phenotype is the death grip in ants infected by fungi within the genus *Ophiocordyceps* [18,19]. These parasites manipulate the behavior of ants to facilitate spore dispersal. Foraging ants encounter fungal spores in the environment, which penetrate the cuticle and start colonization. Once the colony is sufficiently large, the fungus hijacks the central nervous system (CNS), making the ant leave the nest and climb up the foliage where it latches onto the vegetation [18,20]. This death grip involves atrophy of the mandibular muscles leading to a locked jaw preventing the cadaver from falling [19]. Following death, fungal hyphae grow out of the ant, forming a sexual structure from which new spores are dispersed [18]. This manipulated behavior of ants can be traced back to the Eocene with fossil evidence from 48 mya [21]. Such a long co-evolutionary history has resulted in a high specificity, with each infected species of ant examined having its own species of fungus [22–24]. However, not all ant species within a given habitat are infected and previous surveys have shown that ant species that are ecologically and phylogenetically similar to known host species of *Ophiocordyceps* are not necessarily infected [18,20,25–27]. This suggests that barriers to successful infection and behavioral manipulation may exist.

There are many factors that determine which species become hosts to a particular parasite group, such as the encounter rate, host defenses and abiotic factors [28–30]. For a parasite where manipulation of host behavior is necessary for transmission, an important component is the host’s CNS. We would thus expect that manipulators have evolved molecular adaptations to successfully interact with the CNS [10]. Here, we ask how specific such adaptations are. To do this we isolated a newly discovered *Ophiocordyceps unilateralis sensu lato (s.l.)* from infected Carpenter ants found in the temperate woods of South Carolina, USA. Using cultures of this fungal species we tested its ability to manipulate the behavior of four sympatrically occurring ant species: two that are found naturally infected (*Camponotus castaneus* and *Camponotus americanus*) and two (*Camponotus pennsylvanicus* and *Formica dolosa*) that we have not encountered infected despite extensive searches. Since fungi interact with their environment via the secretion of molecules, we focused on the secondary metabolites produced in the presence of ant nervous tissue. To accomplish this we made use of an *ex vivo* ant tissue culturing system combined with metabolomics [31]. By integrating field ecology, lab infections and secondary metabolite discovery, we report that the extended phenotype of brain manipulating fungi can only be expressed in the correct ant host seemingly because the fungus produces a specific array of compounds as a reaction to the presence of the host brain it has evolved to manipulate.

## Results and discussion

### Manipulated dead ant collection and species verification

**Figure 1:**
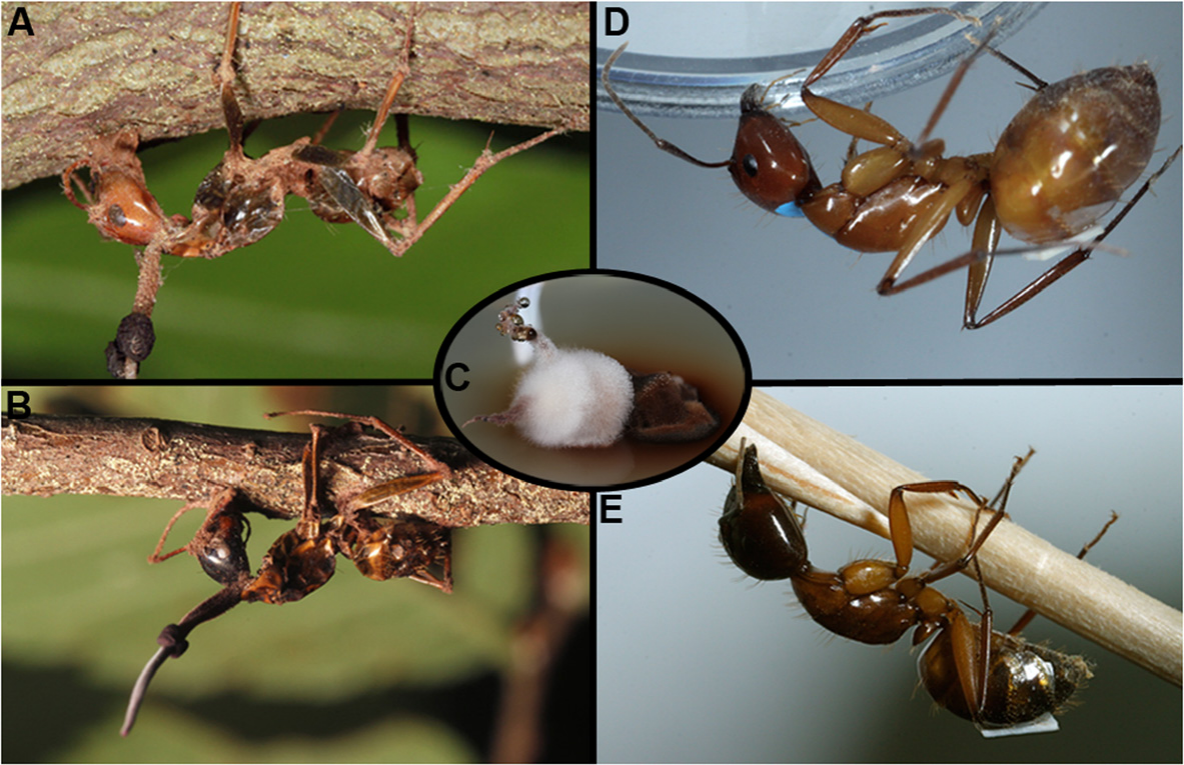
**Natural and lab infections with** ***O. unilateralis s.l.*** **.** **(A-B)** *C. castaneus* **(A)** and *C. americanus* **(B)** infected with *O. unilateralis s.l.* collected in Donalds, SC. **(C)** *O. unilateralis s.l.* culture isolated from an infected *C. castaneus* specimen. **(D-E)** Manipulated *C. castaneus* **(D)** and *C. americanus* **(E)** upon infection with *O. unilateralis s.l.* in the lab.

Previous work on *Ophiocordyceps* in ants has focused on tropical habitats with the consensus that these fungi are uncommon in temperate biomes [26]. However, we discovered a large population of Carpenter ants infected with *O. unilateralis s.l.* in Donald’s County, South Carolina, USA. During our first field survey in 2009 we identified and individually marked 264 fungal killed Carpenter ants (genus *Camponotus*) at one site: 175 *C. castaneus* (Figure 1a) and 82 *C. americanus* (Figure 1b). All ants were attached to twigs from a number of tree species. Position of the death grip was highly stereotyped with 99% of ants attached to the adaxial (lower) side of twigs. Ants were never recorded attached to leaves, which occurs for infected Carpenter ants in tropical habitats [20]. Similar patterns were found in the following years (2010–2013) at this and 5 other sites.

We isolated *O. unilateralis s.l.* from a freshly manipulated and killed *C. castaneus* cadaver (Figure 1c). Species verification was done through *SSU* (small subunit ribosomal RNA gene) sequencing. Blasting the obtained PCR fragment sequence (KJ769099) against all fungi in the NCBI database resulted in a 97% identity with both *O. unilateralis* strain OSC 128574 (DQ522554 [32]) and the very closely related *Ophiocordyceps pulvinata* voucher TNS-F-30044 (GU904208 [24]), verifying that we successfully isolated *O. unilateralis s.l.*.

### Ant infections leading towards species-specific behavioral manipulation

In this study, we addressed if our *O. unilateralis s.l.* species could control the behavior of ants that are not normally found infected in nature. We chose *C. pennsylvanicus* and *F. dolosa,* which we never found infected at our site despite them occurring sympatrically and 1,750 person hours of searching over four years. *C. pennsylvanicus* is within the same genus as the two encountered hosts, *C. castaneus* and *C. americanus*, and is very abundant throughout the East Coast of the USA [33] including South Carolina [34]. *F. dolosa* is an ecologically similar species to *C. castaneus* and *C. americanus* occurring in mixed woodlands with colonies in the soil [35]*.* The genus *Formica* has never been recorded as a host to *O. unilateralis s.l.* [26,36,37]. The two naturally infected ant species were compared to the two naturally uninfected species using infection studies. Since the insect cuticle is a highly heterogeneous structure that varies between species, it is likely to affect the ability of fungal spores to adhere and penetrate the cuticle needed to establish infection [38]. Therefore, we bypassed such external barriers by directly injecting fungal cells into the ants. For each species, 3 replicates of 40 worker ants were placed in a cage containing branches that served as a biting platform and a darkened nest area. Per replicate, 10 ants were injected with fungal cells, 10 were sham treated, and 20 were left untreated. Cages were kept under strict twenty-four hour light and temperature cycles since ant behavior is highly dependent on genes oscillating with circadian rhythms [39–41]. Furthermore, field studies on *O. unilateralis s.l.* infected *Camponotus leonardi* in Thailand, showed synchronization of manipulated biting behavior at solar noon [19]. Next to that, the convergently evolved entomophthoralean fungi are known to cause comparable behavioral manipulations in a range of arthropods (including ants), followed by death and sporulation which also happens at distinct times of day [42–45]. Observations were made 5 times per day for 28 continuous days following infection, recording behavior and time of death for each of the 480 individually marked ants. This set up resulted in the successful reconstruction of behavioral manipulation of ant behavior by a fungal parasite under controlled laboratory conditions.

**Figure 2:**
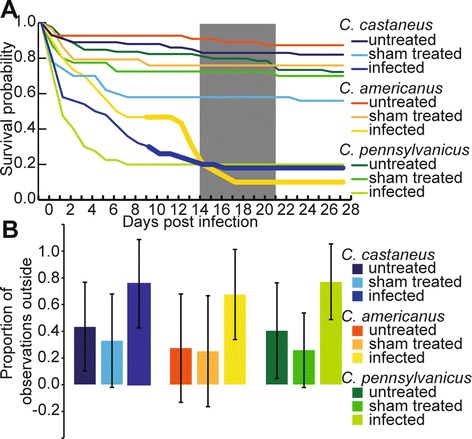
**Survival, fungal growth, and behavioral manipulation of three** ***Camponotus*** **species infected with** ***O. unilateralis s.l.*** **.** **(A)** Kaplan-Meier survival curve for 3 different *Camponotus* species infected with *O. unilateralis s.l.*. The thickened lines represent the time period in which fungal growth was observed. The grey box indicates days post-infection during which behavioral manipulation was observed. **(B)** Mean proportion of observations outside for each species-treatment combination (data presented as mean +/− SD, P<0.001 Tukey post-hoc on two-way ANOVA).

The three *Camponotus* species infected with *O. unilateralis s.l.* had similar survival probabilities (p = 0.475, log-rank test of Kaplan-Meier survival probabilities, Figure 2a). Sham treated and untreated ants for all three species had a significantly higher survival probability than infected individuals (p = 0.00, log-rank test of Kaplan-Meier survival probabilities, Figure 2a). *Formica dolosa* reacted adversely to the injection procedure and was excluded from the analysis. Following death, ant cadavers were monitored for fungal growth emerging from them, which is necessary for parasite transmission. For all three *Camponotus* species no fungal growth was observed upon death <9 days post infection. Fungal growth did emerge from *C. castaneus* and *C. americanus* individuals that died >/=9 days post infection (Figure 2a and Additional file 1a,b). We never observed fungal hyphae emerging from the non-target host cadavers of *C. pennsylvanicus* (Figure 2a). However, fungal blastospores were found inside the bodies of *C. pennsylvanicus* (Additional file 1c,d), demonstrating that the cause of death in these ants was likely due to fungal growth.

In addition, we recorded ant behavior. Infected ants were observed in the foraging arena significantly more often than their healthy and sham-treated nest mates (Tukey post hoc on two-way ANOVA, adjusted p < 0.001 Figure 2b). Only the two species of ants known to be naturally infected (*C. castaneus* and *C. americanus*) were manipulated to die biting (Figures 1d,e, 2a and Additional files 2 and 3), while *C. pennsylvanicus* was never found biting twigs prior to death. Similar to fungal growth, there seems to be a critical period for manipulated biting behavior because we only observed successful manipulation between 14 to 22 days post infection. This implies a parasitic growth phase within the host is needed and that premature death of the host prevents successful fungal development. We suggest that the complex nature of manipulation partially explains why such a long incubation period inside the host is required.

### Metabolomics of species-specific *ex vivo* ant brain–*O. unilateralis s.l.* interactions

Only the two naturally occurring hosts of *O. unilateralis s.l.* could be manipulated despite the ability of the fungus to establish inside all three *Camponotus* species. The lack of manipulation in the non-target ant species, despite the ability of the fungus to kill it suggests that this requires additional factors. The complexity of the manipulated behavior suggests a specific reaction to the host’s CNS is required. This led us to ask if *O. unilateralis s.l.* secretes a different array of metabolites (i.e. displays a heterogeneous secretome) when presented with ant brains of the four different species used in this study. In this experiment, *F. dolosa* has been included again as it allowed us to ask how *O. unilateralis s.l.* would react to ant brains from a different genus. We used a novel protocol that allows the investigation of the secretome of fungal entomopathogens as a reaction to specific insect tissues kept alive *ex vivo* [31]. Through quadrupole time-of-flight mass spectrometry, we measured the unique mass-to-charge/retention time pairs (features) that were significantly enriched in samples where *O. unilateralis s.l.* was grown in the presence of ant brains. Biological replicates for the fungal-brain interaction samples and the various controls (see Methods section) were run together in a randomized order. Within the chosen window of 0.9 and 15 minutes and m/z between 100 and 1100, 37,921 unique ion features were extracted from the raw data (Additional file 4a and [46]).

**Figure 3:**
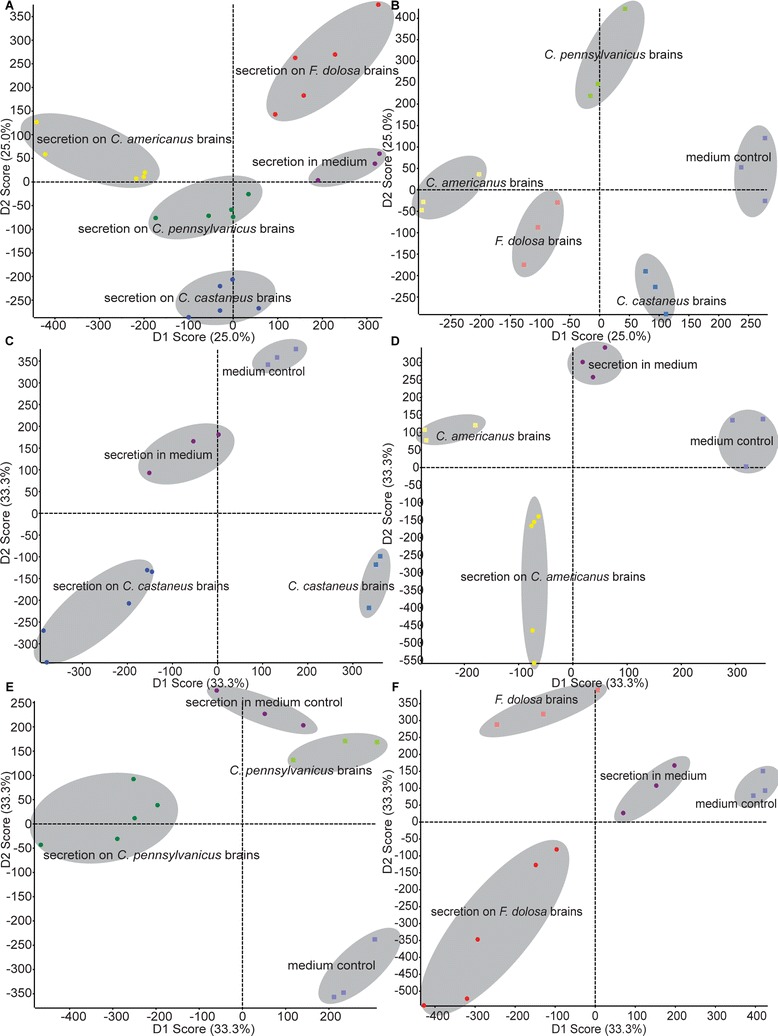
**PCA-DA analyses to determine the heterogeneity of** ***O. unilateralis s.l.*** **on different ant species’ brains.** **(A)** PCA-DA plot showing the clustering of *O. unilateralis s.l.* secretion in the presence of different ant species’ brains kept *ex vivo* in Schneider’s insect medium and the medium without ant brains. **(B)** PCA-DA plot showing the clustering of different ant species’ brains kept *ex vivo* and the medium by itself without fungal growth that served as controls in this study. **(C-F)** PCA-DA plots showing the clustering per species of *O. unilateralis s.l.* secretion in the presence of ant brains of that species versus secretion in the medium without ant brains, ant brains kept *ex vivo* without fungal growth and the Schneider’s insect medium by itself.

**Figure 4:**
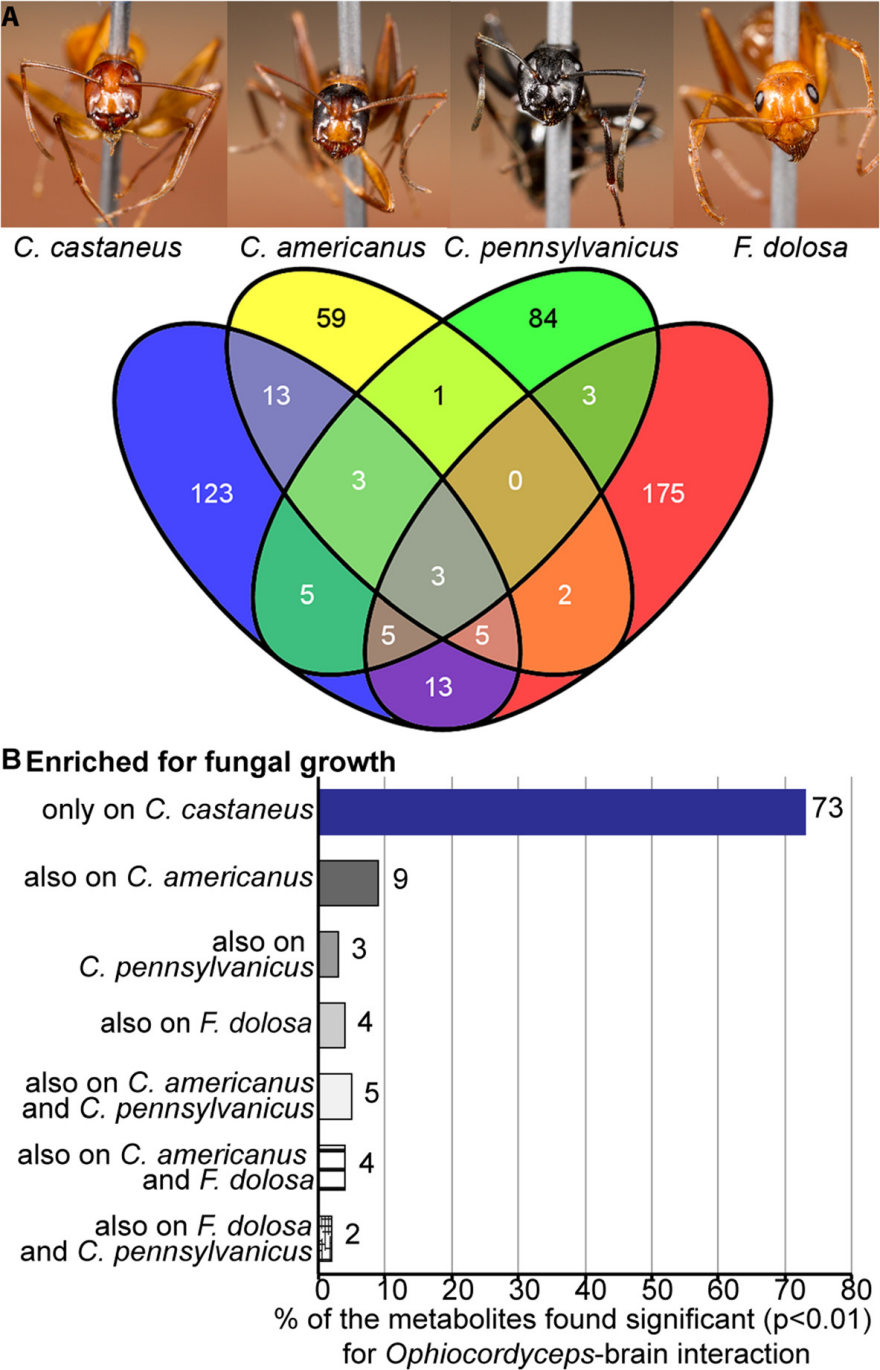
**Heterogeneous metabolite secretion by** ***O. unilateralis s.l.*** **on four ant species’ brains.** **(A)** Venn-diagram comparing all ion features found to be significantly (P < 0.01) enriched in the medium of samples in which *O.unilateralis s.l.* was grown next to *ex vivo* kept ant brains of the species *C. castaneus*, *C. americanus*, *C. pennsylvanicus* and *F. dolosa.* **(B)** Bar chart visualizing how the ions, that were found to be significantly enriched (P < 0.01) due to *O. unilateralis s.l.-C. castaneus* brain interactions across two independent studies, overlap with ions enriched in interactions with other species’ brains.

We performed a principal component analysis and discriminant analysis (PCA-DA, in which discriminating components are calculated with foreknowledge of the samples) on all ion features. This resulted in the clustering of the biological replicates for each species interaction suggesting that, depending on the ant species brains it was presented with, the secretome of *O. unilateralis s.l.* differed (Figure 3a). However, control samples representing the different species’ brains, could also be separated by PCA-DA (Figure 3b). It is therefore possible that the clustering seen for the brain-fungal interactions might be partially due to the brain tissues. This implies that directly comparing *O. unilateralis s.l.* grown beside the brain of one ant species with it grown beside the brain of another is not the best approach when the aim is to specifically study the fungal secretome. Such an approach would lead to the complication of extracting the differences between different species brain tissues from the data set together with the compounds of interest secreted by the fungus as a reaction to those different tissues. Therefore, we performed statistical analyses within a species to rank enriched ion features involved in fungus-brain according to significance, prior to performing an indirect comparison between the species using those ranked ions. Separate PCA-DAs for each of the four species tested resulted in a separate clustering of *O. unilateralis s.l.*–ant brain interaction samples and their corresponding species-specific controls (Figure 3c-f), showing that *O. unilateralis s.l.* reacts to brain tissue through secretion. To rank the ion features enriched as a result of *O. unilateralis s.l.*-ant brain interaction, a t-test for each species was performed in which this interaction was compared to the various controls. Only those ion features that were found to be enriched with a p-value of p < 0.01 were used in the indirect, between species, comparison. We found 170, 86, 104 and 206 significantly enriched molecular weight/retention time peaks for *O. unilateralis s.l.* growth beside *C. castaneus*, *C. americanus*, *C. pennsylvanicus* and *F. dolosa* brains respectively (for feature IDs see Additional file 5). We compared the enriched ion features found for each species interaction, which showed that most of the ion features (between 69% and 85%) were only significantly enriched in one of the four parasite-ant brain interactions (Figure 4a). Our study of the *O. unilateralis s.l.* secretome thus indicates that this fungus reacts heterogeneously to brains from the four ant species we studied by secreting a largely different array of metabolites.

To investigate if these results could be (partly) due to false positives and if our findings hold when the analysis is performed across two independently set up experiments, we repeated the part in which *O. unilateralis s.l.* was grown beside brains of *C. castaneus*, the host species from which we isolated it. In addition, in this set up, the fungus was also grown beside the mandibular muscles of its host. We included this tissue in the analysis to exclude the features that are enriched because of the interaction with ant tissue in general, but are not necessarily brain tissue specific. This resulted in 41,254 unique ion features (Additional file 4b and [46]), which we analyzed using PCA-DA plots that showed clustering of the different biological sample types, as seen before (Additional file 6a-c). Running the samples at the same time and the use of an internal standard in both experiments allowed the comparison of this experiment with the former one. The samples of this second experiment were analyzed together with the *C. castaneus* related samples of the first one (Additional file 4c, Additional file 6d). To rank the ion features that were significantly enriched as a reaction to brain tissue, again a t-test was performed in which the fungus-brain interaction was compared to the various controls. Choosing again a cut off of p < 0.01 we found 258 significantly enriched ion features within the secretome of *O. unilateralis s.l.* grown on *C. castaneus* brains (Additional file 7a). Comparing these enriched ion features with those found to be enriched for *O. unilateralis s.l.-C. castaneus* brain interactions in first analysis described above, 56 were found to be in common (listed in Additional file 8). The discovery of these significantly enriched ion features across two independent experiments suggests that these are biologically relevant and not false positives due to the experimental set up or data mining. We compared these 56 ion features that *O. unilateralis s.l.* produced in the presence of *C. castaneus* brains again with the ion features found to be enriched for *O. unilateralis s.l.* growth in the presence of other ant species brains. Of these ion features, 73% were only significantly enriched in the presence of *C. castaneus* brains (Figure 4b). This more conservative analysis of the data thus resulted in the same conclusion that *O. unilateralis s.l.* secretes a specific set of metabolites depending on the ant brain it encounters. In addition to providing insight into the patterns of specificity of organisms evolved to control brains, these experiments resulted in candidate compounds that might be involved in establishing behavioral manipulation.

### Identification of candidate metabolites involved in brain manipulation

Our attempt to identify metabolites focused on the ion features enriched in the secretome of *O. unilateralis s.l.* in the presence of brains of its natural host *C. castaneus*. We found 56 ion features across two experiments (Additional file 8). In an attempt to identify these we compared their MS/MS product ion mass spectra with those of a similar m/z value in the METLIN database [47]. A present roadblock is however that metabolite databases do not yet hold an extensive amount of mass spectra and do not cover all eukaryote species to the same extent. Therefore, in most of the cases where a MS/MS product ion scan was generated, no match was found. This of course results in limitations that are inherent to the use of metabolomics in a study like the one presented here, but with more data available, this will improve over time. Despite this, we did manage to putatively identify one of the candidate ion features to be guanidinobutyric acid (GBA, m/z 146.0914 at 1.08 min., 2.6 fold higher in the secretome of *O. unilateralis s.l.* grown in the presence of *C. castaneus* brains, Additional file 7a). This identification was verified by comparing the retention time and product ion MS/MS spectra obtained from our samples with an authentic standard (CAS 463-00-3, Sigma Aldrich; Additional file 9a). GBA is involved in the transport of compounds such as creatine and guanidinoacidic acid (GAA) across the blood–brain barrier [48] and known to be involved in epileptic discharges and convulsions in rodents [49]. Altered levels of creatine and GAA have been shown to cause neurological disorders [50]. GBA has also previously been isolated from the fungus *Trogia venenata,* which has been implicated in causing sudden deaths in Yunnan, China [51].

To search for additional compounds we adopted a less stringent but still significant cut off value of p < 0.05 for our ranking analysis across the two experiments. This returned an additional 1038 ion features, which still comprises only the top 2.6% of all ion features from the secretome of *O. unilateralis s.l.* grown beside the brains of *C. castaneus* (Additional file 7b). Among these features we putatively identified a sphingosine (m/z 300.2891 at 12.96 min., 2.6 fold higher in the secretome of *O. unilateralis s.l.* grown in the presence of *C. castaneus* brains, Additional file 7b). The identified metabolite was verified by comparing the retention time and product ion mass spectrum to that of an authentic standard of L-threo-sphingosine (CAS 25695-95-8, Cayman Chemical; Additional file 9b). Sphingosines are part of sphingolipid metabolism, which affects all types of cell regulation [52,53]. Defects can lead to cancers [54] and neurological syndromes [55]. The secretion of fungal derived sphingosines have not been reported to this date, but several different fumonisins, that mimic compounds involved in sphingolipid metabolism, have been [56]. These compounds are produced by several plant pathogenic *Fusarium* species growing on cereals causing leukoencephalomalacia (‘hole in the head disease’) in life stock being fed with infected crops [57]. We examined our metabolomics dataset for these fumonisins but did not find any.

We identified two compounds that were enriched in the secretome of *O. unilateralis s.l.* grown beside *C. castaneus* brains. Since these compounds (GBA and sphingosine) have known neurological effects on mammals [49,50,55] we tested their effect on ants. We injected serial dilutions of the authentic standards of GBA and L-threo-sphingosine separately and in combination into ants of the species *C. castaneus*. This however did not result in behavioral effects similar to those induced by *O. unilateralis s.l.* infection. It is likely that the injection of specific candidate metabolites is not biologically realistic because based on our profiling of the fungal secretome we expect that multiple compounds, and therefore mechanisms, act in concert. This is in line with the conclusions that can be drawn from studies in *T. gondii* [14–17], described in the Background section.

## Conclusions

We assessed the species-specificity of ant brain manipulation by the specialized fungus *O. unilateralis s.l.*. The fungus was isolated from a manipulated, twig-biting cadaver of the ant species *C. castaneus* in the temperate woods of South Carolina, USA. Cultures of this fungus were used to develop an infection protocol in which ant hosts are infected through an injection of fungal material. This lead to the major advancement of lab-induced behavioral manipulation of an insect by a fungal parasite, opening up new possibilities to study the many different, complex aspects of parasite-host interactions during behavioral manipulation. These aspects range from integrative studies looking into social behavior, to host-parasite co-evolution, disease dynamics, host immunology and molecular mechanisms employed by the parasite.

Here, we used this technique to investigate species-specificity beyond external factors such as life history, environment and cuticle compatibility for spore attachment. We conclude from these studies that *O. unilateralis s.l.* is able to kill different species of ants, including species that are not found infected in nature. However, the fungus is not able to manipulate the behavior of all of them, which is crucial for successful transmission. Only the two species tested that are also manipulated by *O. unilateralis s.l.* in nature, displayed the characteristic biting behavior prior to death in the lab. This suggests that the fungus reacts heterogeneously to ant brains of different species causing it to be able to alter the activity of the CNS of some, but not all ants. Despite a growing interest in parasitic behavioral manipulation, the mechanisms and compounds used by parasites to alter CNS activity are still largely unknown. In this study we move towards a better understanding of this phenomenon through the discovery of candidate compounds *O. unilateralis s.l.* employs to establish ant brain manipulation while simultaneously testing our hypothesis that it employs different compounds in different host species. We therefore looked at the metabolites this fungus secretes as a reaction to different ant species brains. Metabolomics analysis on an *ex vivo* system showed that *O. unilateralis s.l.* indeed reacts heterogeneously to the brains of different ant species. Furthermore, our experiments resulted in the discovery of candidate manipulator compounds, which had not been reported before for this specialist entomopathogen. Despite metabolite discovery being a challenging and difficult endeavor we succeeded in identifying two candidate metabolites that, based on their known effects, are likely part of the complex mechanisms underlying brain manipulation. We found that guanobutyric acid (GBA) and sphingosine, both reported to be involved neurological disorders, were enriched when the fungus was grown in the presence of brains of its target species. However, since both compounds are present in animal cells, the possibility exists that their significant enrichment is the result of other yet to be identified fungal compounds causing higher levels of GBA and sphingosine to be released from the brain. GBA has however also been reported to have been isolated from fungal material, and though the secretion of fungal derived sphingosines have not been reported, fungal toxins, that mimic compounds involved in sphingolipid metabolism, have been. This implies that, if the increased sphingosine and GBA levels are indeed caused by *O. unilateralis s.l.* secreting them as a reaction to ant brain tissue, the fungus might be producing more compounds that are similar to the ones the host produces. Future fungal genome and transcriptome studies, combined with a better understanding of ant brain physiology, will help to fully determine the undoubtedly complex mechanisms at play in this intriguing example of behavioral manipulation.

## Methods

### Specimens collection and fungal culturing

We sampled and recorded data on *O. unilateralis s.l.* infected *C. castaneus* and *C. americanus* in Donald’s, South Carolina, USA (GPS 34.375215, −82.346937) between December 2009 and July 2013. With one of us living at the field site, spending at least 1 hour a day in 2009 and 2010, and 3 lab members visiting it yearly for 2 weeks since 2011, +/− 1750 man hours were spent collecting data. The *O. unilateralis s.l.* species used in this study was isolated from an infected *C. castaneus* ant from this area. The specimen was surface sterilized with ethanol, dissected and fungal material was placed in petri dishes containing 1/5 potato dextrose agar (Himedia), 3/5 water agar (Alfa Aesar), 100 μg/mL kanamycin (Invitrogen) and 100 units/mL penicillin and 100 μg streptomycin/mL (Invitrogen). When fungal growth was observed, fungal plugs were transferred to rich liquid medium consisting of Grace’s insect medium (Sigma) and 10% fetal bovine serum (PAA Laboratories Inc.). Colonies used for infections were transferred back to PDA plates prior to colony disruption.

### DNA extraction, PCR and sequencing

To confirm the isolate was *O. unilateralis s.l.*, small subunit (*SSU*) was amplified, sequenced, and blasted using the Megablast algorithm against the entire Fungi (taxid:4751) nucleotide collection (nr/nt) (blastn, NCBI). Genomic DNA extraction was performed on flash frozen fungal mycelium mechanically disrupted inside a frozen 2 mL eppendorf with two 5/32 inch metal balls (Wheels Manufacturing Inc.) using a TissueLyser II (Qiagen) and a chilled adapter set (Qiagen) at 24 freq/sec for 60 sec. DNA was taken up in 900 μL extraction buffer (1% SDS, 155 mM 4-aminosalicyclic acid and 0.2 mL/mL 5× RNB (1.0 M Tris, 1.25 M NaCl, 0.25 M EGTA, pH 8.5)), and 900 μL phenol:chloroform:isoamyl alcohol 25:24:1. After centrifugation for 10 min. at 10.000 × g the water phase was taken up in 5 volumes PB buffer (Qiagen) and the DNA was purified using the columns, reagents and protocol of the QIAquick PCR Purfication Kit (Qiagen). *SSU* was amplified with primers NS1 and NS4 [58] using Platinum Taq DNA polymerase (Invitrogen), reaction conditions advised by the manufacturer of the enzyme, and the cycler program described in [59]. The PCR product was sequenced by Macrogen (Maryland, USA) with the same primers for initial amplification.

### Infections

To infect ants with *O. unilateralis s.l.*, fungal colonies were placed in a sterile 2 mL tube with two 8/32 inch metal balls (Wheels Manufacturing Inc.) and 500 μl Grace’s medium (Sigma) freshly supplemented with 10% FBS (PAA laboratories Inc.). The colony was disrupted using a TissueLyser II (Qiagen) at RT for 60 sec. at 30 freq/sec. This way single hyphae were obtained that were used at a mean concentration of 3.9×10^7^ +/− 1.1×10^7^ hyphae/ml. Infections were done by injecting 1 μL hyphal solution with a laser pulled 10 μL micropipette (Drummond) and aspirator tube (Drummond) into the thorax underneath the front legs. Sham treatments were done in similar fashion using 1 μL medium without hyphae. To test the effect of the two candidate compounds identified in this work, commercially obtained standards were injected alone or in combination using the same method. Serial 10 times dilutions between 1 μg/ml and 100 pg/ml were made in Grace’s medium with FBS. For the combination both equal concentrations and a series of dilutions in opposite directions were combined (100 μg/ml sphingosine with 100 pg/ml GBA, 10 μg/ml sphingosine with 1 ng/ml GBA, etc.). Since sphingosine was dissolved in ethanol, shams were injected with ethanol concentrations between 10% and 0.00001%.

### Ant species and behavioral observations

Workers of four different ant species were used: *C. castaneus*, *C. americanus*, *C. pennsylvanicus* and *F. dolosa*. Colonies were obtained in SC, USA (private properties in Due West (GPS 34.332110, −82.387131), Donalds (GPS 34.375215, −82.346937) and Greenville (GPS 34.844313, −82.385428)) and PA, USA (Shaver’s Creek (GPS 40.6667, −77.9098)). Each replicate was housed in a 513 cm^2^ cage with sand and a darkened, two-dimensional 140 cm^2^ wooden nest. Ants were given *ad libitum* water and 10% sugar water and a climbing and biting set up made of foam embedded with toothpicks and twigs. A strict light and temperature cycle was maintained with light between 06:00 h and 18:00 h and a 10°C increased temperature between 10:00 h and 16:00 h. In each of the 3 replicates per ant species, 40 ants were individually labeled with unique color-coding combinations (Edding). Ants were randomly assigned to a treatment group: 10 infected, 10 sham treated and 20 untreated. Observations were made every day, with 3 hour intervals from 9:00 h to 21:00 h. The survival status, location and any unusual behaviors (i.e. twig biting) were recorded. Survival data was analyzed using log-rank tests on Kaplan-Meier survival probabilities (R version 2.15.1, ‘survival’ package). A post-hoc Tukey on a two-way ANOVA was conducted to determine the effect of species and treatment on the mean proportion of observations in which ants were outside in the foraging arena. Upon death, ants were removed, surface sterilized, and placed in a humid environment at 28°C for up to 7 days to monitor emerging fungal growth. Additionally, *C. pennsylvanicus* cadavers were dissected in PBS to determine fungal development within the body. Haemolymph from the abdomen was mounted in 0.1% fuchsin (lacto fuchsin (Sigma) in lactic acid (Fluka)) and observed using an Olympus BX41 light microscope with 20× and 40× lenses and fitted with an Olympus U-TV1x-2 and U-CMAD3 imaging camera and PictureFrame™ Application 2.2 software.

Metabolite injections were done in a similar cage set ups using 2 *C. castaneus* ant colonies. For each condition 2 ants were injected and uniquely color-coded. Behavior was monitored for 7 days, including day of injection.

### *Ex vivo* insect tissue culturing

Ant brains were dissected in PBS from the four ant species heads using a dissecting scope (Olympus SZX16) and the methods described in [31]. Tissues were washed three times and placed in 1 mL Schneider’s containing FBS and antibiotics for 3 days incubation at 28°C. To measure metabolites secreted by *O. unilateralis s.l.*, as a reaction to the ant tissues, to each of the five biological replicates a colony of 3 mm diameter was added per single cell culture insert. Controls were incorporated in triplicate by incubating fungal material without ant brains, ant brains without fungal material, and the medium without fungal material or ant brains. A similar set up was used in the second experiment in which the metabolites secreted by *O. unilateralis s.l.* on *C. castaneus* brains were again compared versus all controls mentioned above, plus mandibular muscle tissue from the ant’s heads.

### Metabolomics

To measure secreted metabolites, the medium was harvested and 100 μl was mixed with 1 volume of acetonitrile for protein precipitation prior to LC-MS/MS analysis. The acetonitrile was supplemented with 1 μM chlorpropamide standard (Santa Cruz Biotechnology, m/z 277.0408 at 10.12 [10.081-10.163] minutes) to align the raw data from all samples in this study. Samples were randomized and run using the HPLC-QTOFMS (Shimadzu Prominence UFLC XR and AB Sciex 5600 quadrupole time-of-flight mass spectrometry) platform. Samples (5 μl) were separated on a C18 Column (100 × 2.1 mm 1.7 um, Waters Acquity BEH) using a gradient elution program with aqueous acetonitrile (3-90%) at a flow rate of 250 ul/min. Positive ion electrospray ionization mass spectra were acquired over the mass range 50–1250 Da in IDA (Information Dependent acquisition) mode with one 100 ms survey scan and up to twenty 100 ms MS/MS product ion scans per duty cycle. The survey scan LC-MS raw datasets were aligned together using MarkerView (AB Sciex) with the following parameters: Retention times between 0.00 and 15.00 min.; Subtraction Offset of 10 scans; Subtraction Mult. Factor of 1.3; Noise Treshold of 50; Min. Spectral Peak Width of 15 ppm; Min. RT Peak Width of 3 scans; Retention Time Tolerance of 0.20 min.; Mass Tolerance of 20.0 ppm; 3 required samples; Maximum of 100000 ion features; performed RT correction using the chlorpropamide standard; performed normalization of the data. Analyses were done on all ion features with an m/z between 100 and 1100 and a retention time between 0.9 and 15 minutes with the MarkerView (AB Sciex) software package. This software was subsequently used to analyze the data using supervised principle component analyses (PCA-DA) and t-tests to rank the ion features from most to least significantly enriched due to the fungal parasite-host brain interaction. In this test, per species, the ion features present in fungal-ant brain samples were compared to features present in all controls: 1) the background medium, 2) fungal growth in the medium, and 3) ant brains kept *ex vivo* in the medium.

Putative compound identification was done by comparison of the MS/MS product ion mass spectra with those of a similar m/z value (+/− 5 ppm) in the METLIN database [47]. We used PeakView (AB Sciex) to examine the raw data files [46], for the accurate m/z values of the features. Putatively identified ion features were verified by running their commercial standards together with samples found to be positive (*O. unilateralis s.l.* with *C. castaneus* brains) for these metabolites.

### Availability of supporting data

The raw mass spectrometry data sets supporting the results of this article are available in the metabolomics.psu.edu repository, http://dx.doi.org/10.13014/D3KW5CXX.

The other data sets supporting the results of this article are included within the article (and its additional files).

## Additional files

Additional file 1
**Fungal growth emerging from and inside** ***Camponotus*** **species infected with** ***O. unilateralis s.l.*** **.** (A-B) Fungal growth emerging from *C. castaneus* (A) and *C. americanus* (B) cadavers upon injection with *O. unilateralis s.l.* 9+ days after infection. (C-D) Microscopic pictures at a magnification of 20x (C) and 40x (D) of fungal blastospores observed in the abdomens of *C. pennsylvanicus* cadavers that were injected with *O. unilateralis s.l.* hyphal material. No fungal growth was observed emerging from *C. pennsylvanicus* cadavers. Additional file 2
**Manipulated biting behavior in** ***C. castaneus.*** Video recording of manipulated biting behavior seen in *C. castaneus.*
Additional file 3
**Manipulated biting behavior in** ***C. americanus.*** Video recording of manipulated biting behavior seen in *C. americanus.*
Additional file 4
**Datasheets containing unique features found in this study.** Sheet a: all unique retention time / mass to charge ratio peaks that are found between 100 and 1100 m/z with retention times between 0.9 and 15 minutes of samples where *O. unilateralis s.l.* was grown in the presence of 4 different species’ ant brains, growth of the fungus in the supplemented insect medium that was used in this study, and the 4 species’ ant brains kept by themselves in said medium. Sheet b: all unique retention time/mass to charge ratio peaks that are found between 100 and 1100 m/z with retention times between 0.9 and 15 minutes of samples where *O. unilateralis s.l.* was grown in the presence of *C. castaneus* ant brains or muscles, growth of the fungus in the supplemented insect medium that was used in this study, and the *C. castaneus* ant brains and muscles kept by themselves in said medium. Sheet c: all unique retention time / mass to charge ratio peaks that are found between 100 and 1100 m/z with retention times between 0.9 and 15 minutes for all *C. castaneus* related samples and controls in this study. Additional file 5
**Enriched features in** ***O. unilateralis s.l.*** **– different ant species’ brains interactions.** All unique m/z /retention time peaks found to be higher with a p-value <0.01 in samples where *O. unilateralis s.l.* was grown on 4 different species’ ant brains compared to all controls for that ant species. Additional file 6
**PCA-DA plots to determine tissue specificity of** ***O. unilateralis s.l.*** **on** ***C. castaneus*** **tissues.** (A) PCA-DA plot showing the clustering of *O. unilateralis s.l.* secretion in Schneider’s medium, medium with ant brains and medium with mandibular muscles. (B-C) PCA-DA plots showing the clustering of *O. unilateralis s.l.* in the presence of (B) ant brains or (C) ant mandibular muscles versus their respective tissue controls, the medium control and fungal growth in the medium without ant tissues. (D) PCA-DA plot showing the clustering of all sample types related to *O. unilateralis s.l.* secretion in the presence of *C. castaneus* tissues that were generated across the two metabolomics data sets discussed in this study. Additional file 7
**Enriched features in** ***O. unilateralis s.l.*** **–** ***C. castaneus*** **brains interactions across 2 independent experiments.** Sheet a: All unique m/z/retention time peaks found to be higher with a p-value <0.01 in samples across two independent experiments where *O. unilateralis s.l.* was grown on *C. castaneus* brains compared to all controls for that ant species. Sheet b: All unique m/z/retention time peaks found to be higher with a p <0.05 in samples across two independent experiments where *O. unilateralis s.l.* was grown on *C. castaneus* brains compared to all controls for that ant species. Additional file 8
**Commonality between ant species of enriched features found across 2 independent studies.** p < 0.01 peaks found in common for the independent biological experiments done in this study and the separate analyses of both experiments. Peaks are split up in groups as illustrated in the bar chart of Figure 4b. Additional file 9
**Mass spectra of the two candidate metabolites likely involved in brain manipulation by** ***O. unilateralis s.l.*** **identified in this study.** (A) Mirror image of the guanidinobutyric acid found in *O. unilateralis s.l.*-*C. castaneus* brain interaction samples and a standard for 4-guanidinobutyric acid (CAS 463-00-3, Sigma Aldrich) at a concentration of 50 μg/mL (in red). (B) Mirror image of the identified sphingosine found in *O. unilateralis s.l.*-*C. castaneus* brain interaction samples and a standard for L-threo-sphingosine C-18 (CAS 25695-95-8, Cayman Chemical) at a concentration of 1 μg/mL (in red).
