# Supplementary figures and images for: Species-specific ant brain manipulation by a specialized fungal parasite
Source: BMC Evol Biol. 2014 Aug 29;14:166. doi: 10.1186/s12862-014-0166-3 (PMC4174324; doi:10.1186/s12862-014-0166-3)

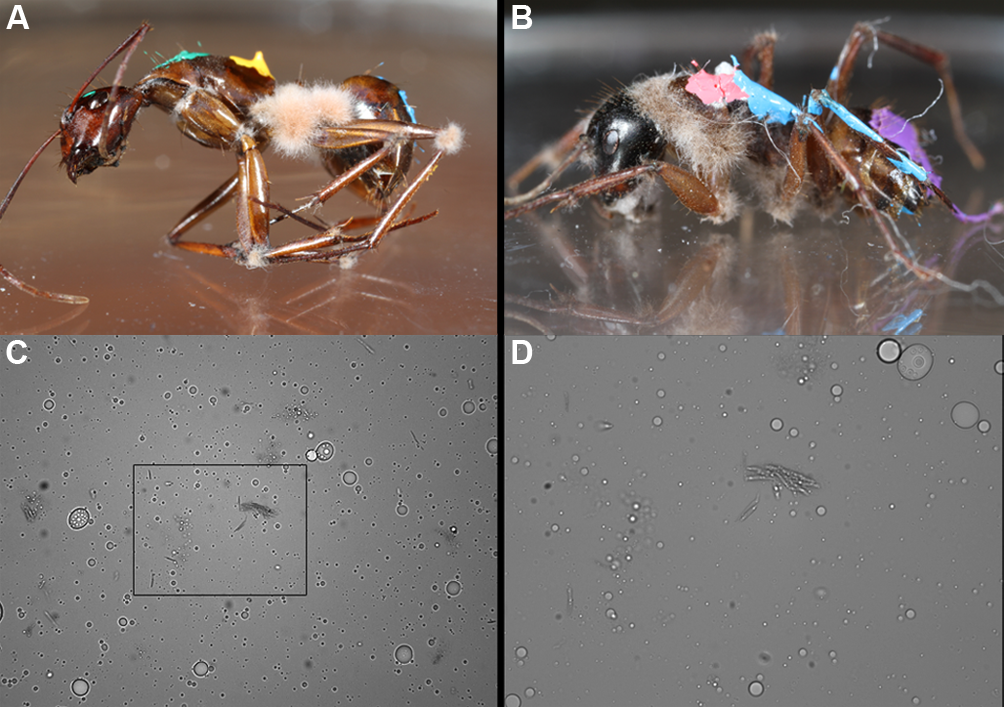

Supplement: Additional file 1 — Fungal growth emerging from and inside Camponotus species infected with O. unilateralis s.l. . (A-B) Fungal growth emerging from C. castaneus (A) and C. americanus (B) cadavers upon injection with O. unilateralis s.l. 9+ days after infection. (C-D) Microscopic pictures at a magnification of 20x (C) and 40x (D) of fungal blastospores observed in the abdomens of C. pennsylvanicus cadavers that were injected with O. unilateralis s.l. hyphal material. No fungal growth was observed emerging from C. pennsylvanicus cadavers. [file 12862_2014_166_MOESM1_ESM.tiff]

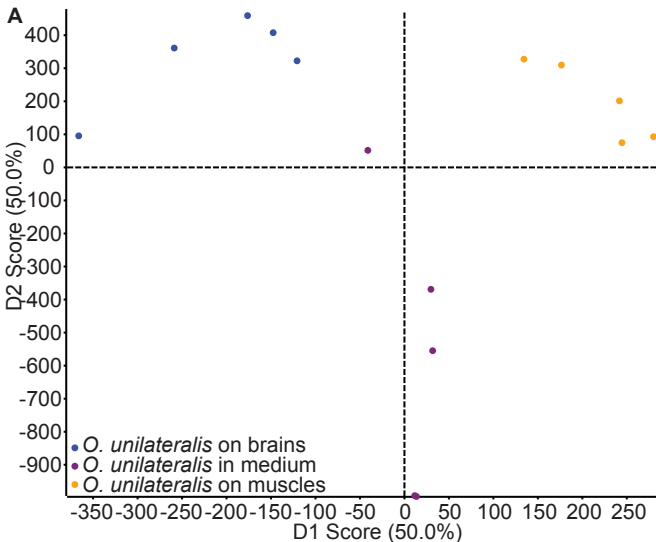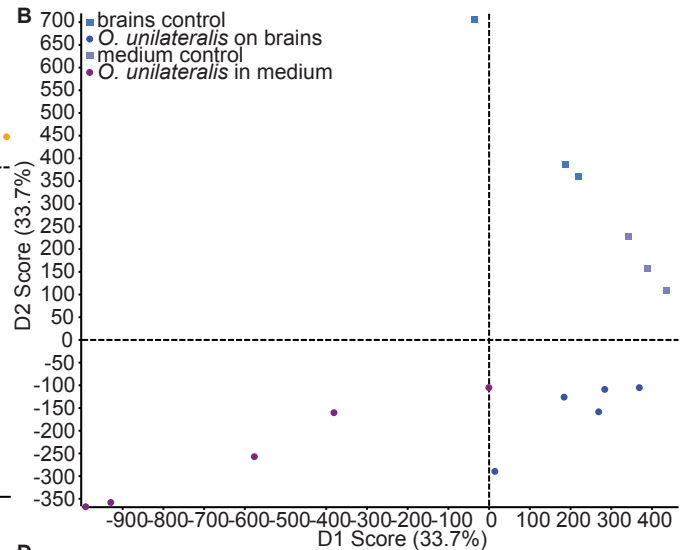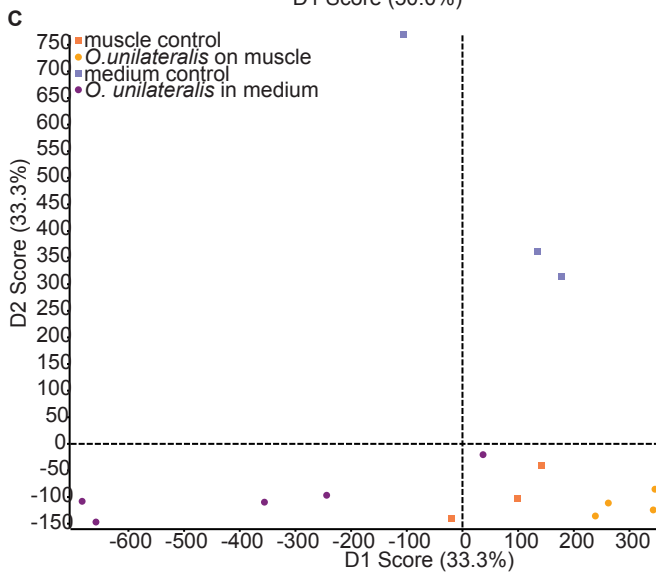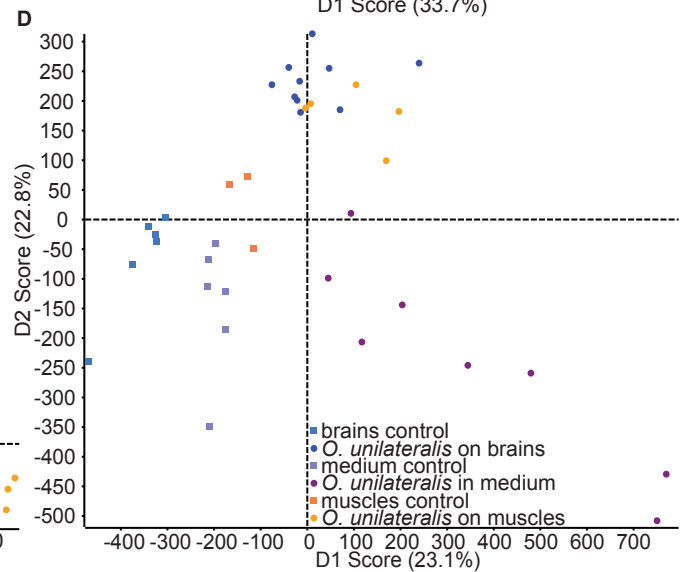

Supplement: Additional file 6 — PCA-DA plots to determine tissue specificity of O. unilateralis s.l. on C. castaneus tissues. (A) PCA-DA plot showing the clustering of O. unilateralis s.l. secretion in Schneider’s medium, medium with ant brains and medium with mandibular muscles. (B-C) PCA-DA plots showing the clustering of O. unilateralis s.l. in the presence of (B) ant brains or (C) ant mandibular muscles versus their respective tissue controls, the medium control and fungal growth in the medium without ant tissues. (D) PCA-DA plot showing the clustering of all sample types related to O. unilateralis s.l. secretion in the presence of C. castaneus tissues that were generated across the two metabolomics data sets discussed in this study. [file 12862_2014_166_MOESM6_ESM.pdf]

A

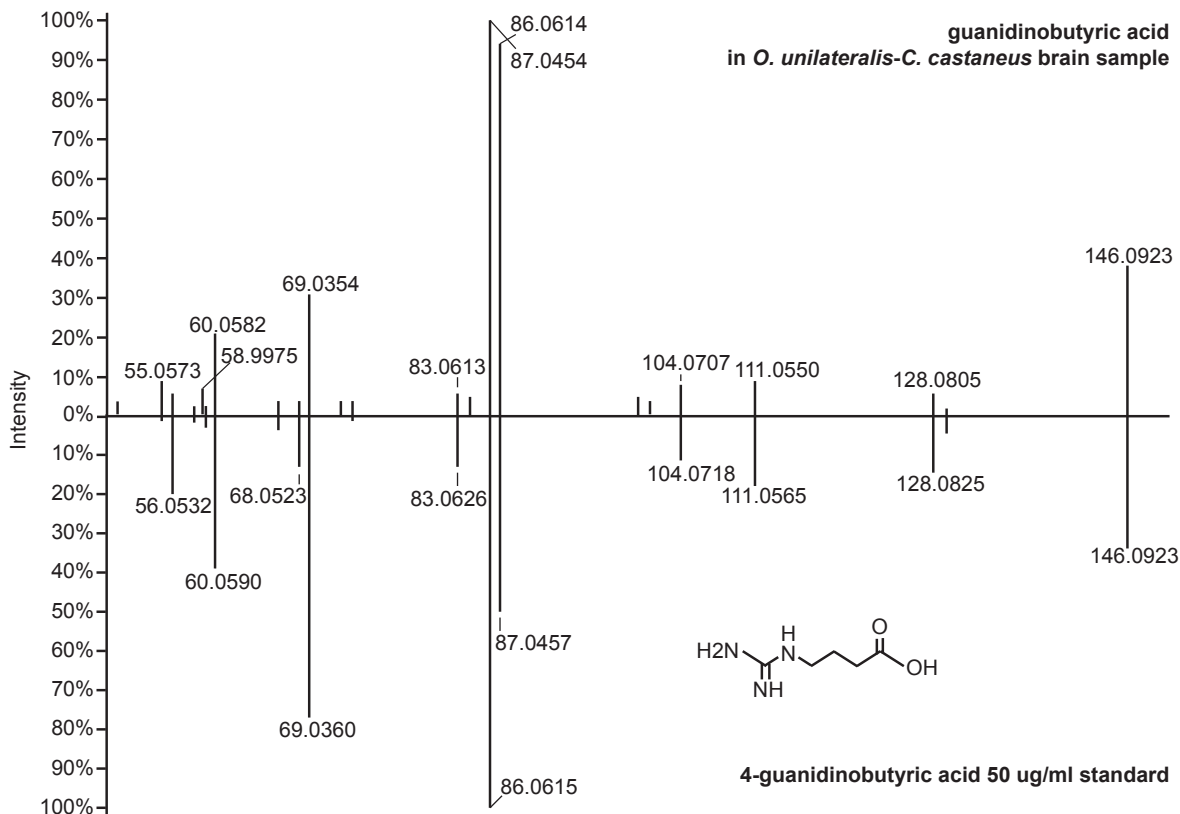

B

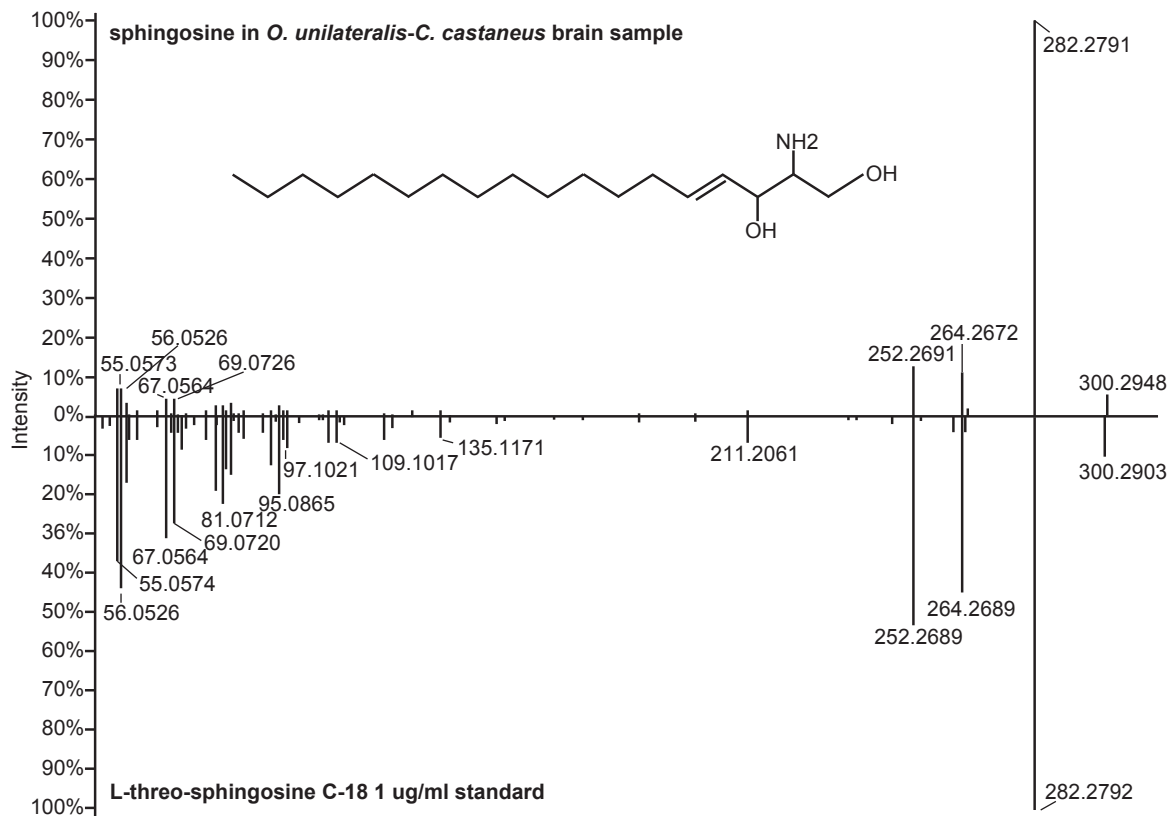

Supplement: Additional file 9 — Mass spectra of the two candidate metabolites likely involved in brain manipulation by O. unilateralis s.l. identified in this study. (A) Mirror image of the guanidinobutyric acid found in O. unilateralis s.l.-C. castaneus brain interaction samples and a standard for 4-guanidinobutyric acid (CAS 463-00-3, Sigma Aldrich) at a concentration of 50 μg/mL (in red). (B) Mirror image of the identified sphingosine found in O. unilateralis s.l.-C. castaneus brain interaction samples and a standard for L-threo-sphingosine C-18 (CAS 25695-95-8, Cayman Chemical) at a concentration of 1 μg/mL (in red). [file 12862_2014_166_MOESM9_ESM.pdf]
